# Effect of a novel bioceramic root canal sealer on the angiogenesis-enhancing potential of assorted human odontogenic stem cells compared with principal tricalcium silicate-based cements

**DOI:** 10.1590/1678-7757-2019-0215

**Published:** 2019-12-17

**Authors:** Keziban Olcay, Pakize Neslihan Taşli, Esra Pamukçu Güven, Gül Merve Yalçın Ülker, Emine Esen Öğüt, Elif Çiftçioğlu, Binnur Kiratli, Fikrettin Şahin

**Affiliations:** 1 Istanbul Medipol University, Faculty of Dentistry, Department of Endodontics, Istanbul, Turkey.; 2 Yeditepe University, Faculty of Engineering and Architecture, Department of Genetics and Bioengineering, Istanbul, Turkey.; 3 Istanbul Okan University, Faculty of Dentistry, Department of Endodontics, Istanbul, Turkey.; 4 Istanbul Okan University, Faculty of Dentistry. Department of Oral and Maxillofacial Surgery, Istanbul, Turkey.; 5 Istanbul Medipol University, Faculty of Dentistry, Department of Oral and Maxillofacial Surgery, Istanbul, Turkey.

**Keywords:** Angiogenesis inducing agents, Dental cements, Regenerative endodontics, Stem cells

## Abstract

**Objective:**

This study evaluated the angiogenesis-enhancing potential of a tricalcium silicate-based mineral trioxide aggregate (ProRoot MTA), Biodentine, and a novel bioceramic root canal sealer (Well-Root ST) in human dental pulp stem cells (hDPSCs), human periodontal ligament stem cells (hPLSCs), and human tooth germ stem cells (hTGSCs).

**Methodology:**

Dulbecco's modified Eagle's medium was conditioned for 24 h by exposure to ProRoot MTA, Biodentine, or Well-Root ST specimens (prepared according to the manufacturers’ instructions). The cells were cultured in these conditioned media and their viability was assessed with 3-(4,5-dimethyl-thiazol-2-yl)-5-(3-carboxy-methoxy-phenyl)-2-(4-sulfo-phenyl)-2H tetrazolium (MTS) on days 1, 3, 7, 10, and 14. Angiogenic growth factors [platelet-derived growth factor (PDGF), basic ﬁbroblast growth factor (FGF-2), and vascular endothelial growth factor (VEGF)] were assayed by sandwich enzyme-linked immunosorbent assay (ELISA) on days 1, 7, and 14. Human umbilical vein endothelial cell (HUVEC) migration assays were used to evaluate the vascular effects of the tested materials at 6–8 h. Statistical analyses included Kruskal–Wallis, Mann–Whitney U, and Friedman and Wilcoxon signed rank tests.

**Results:**

None of tricalcium silicate-based materials were cytotoxic and all induced a similar release of angiogenic growth factors (PDGF, FGF-2, and VEGF) (p>0.05). The best cell viability was observed for hDPSCs (p<0.05) with all tricalcium silicate-based materials at day 14. Tube formation by HUVECs showed a significant increase with all tested materials (p<0.05).

**Conclusion:**

The tricalcium silicate-based materials showed potential for angiogenic stimulation of all stem cell types and significantly enhanced tube formation by HUVECs.

## Introduction

Guided endodontic repair refers to regenerative therapies that have as their first priorities: periapical lesions healing, root development promotion, root canal walls thickening, and apical foramen maturation induction to maintain dental pulp vitality. These steps in the repair process are essential to ensure the repaired teeth durability and functionality.

Wound healing and repair depend on angiogenesis to promote neovascularization.^1^ The angiogenic response is controlled by the cumulative effects of positive and negative regulatory factors.^2^ In particular, a role for a number of polypeptide growth factors has been identified in the initiation of the angiogenic response and regulation of endothelial cell proliferation in wound healing.^3^ These factors include basic ﬁbroblast growth factor (FGF-2), platelet-derived growth factor (PDGF), and vascular endothelial growth factor (VEGF; which is also designated as vascular permeability factor and ﬁbroblast growth factor). VEGF is considered essential for the vascular system differentiation.^4^ Similarly, FGF-2 stimulates new blood vessels growth and development (angiogenesis) that contribute to normal wound healing and tissue development^5^ and plays a signiﬁcant role in the neovascularization of damaged or traumatized tissue,^6^ whereas PDGF functions in tissue regeneration and embryogenesis. VEGF production also provides important information regarding cells functionality.^7^ Scientific literature indicates that these growth factors possibly participate in the angiogenic response of the dental pulp and periapical tissues; therefore, their role in regenerative or vital pulp therapies needs further exploration. In this respect, a key goal of relevant research should be to discern the interaction between bioactive endodontic materials and the growth factors released during regeneration and/or revascularization, as well as their effects on the angiogenic responses of adjacent tissues.

Guided endodontic repair has been conducted for many years in Dentistry using mineral trioxide aggregate (MTA) and other bioactive endodontic materials.^8^^,^^9^ MTA has been recognized as the approved gold standard in guided endodontic repair therapies for many years because of its capacity to induce smooth hard tissue deposition with low pulpal inflammation^10^ and for its biocompatibility on cells regarding its reparative, regenerative, and angiogenic effects.^11^^,^^12^ However, novel tricalcium silicate-based cements, such as Biodentine (Septodont,. Saint-Maur-des-Fossés, France), have recently been introduced to overcome the somewhat intolerable drawbacks of MTA, such as its long setting time,^13^ difficult handling properties,^13^ and tooth discoloration.^14^

Biodentine was produced using active biosilicate technology to serve as a bioactive dentin substitute.^13^ The mixture is prepared in a powder-to-liquid form in a single-dose capsule, to be mixed with an amalgamator for 30 s. The cement is then applied to the cavity as a bulk dentin substitute without any requirement for adhesive technology.^15^ The calcium chloride content of Biodentine leads to a much shorter setting time (12 min) than ProRoot MTA (3-4 h), superior handling characteristics, and enhanced angiogenic and osteogenic capacity when administered to human mesenchymal stem cells.^12^ These beneficial factors support the Biodentine use as an agreeable alternative bioactive material for use in guided endodontic repair.

One recently developed alternative is Well-Root ST (Vericom, Gangwon-Do, Korea), a premixed, ready to use, and injectable bioactive root canal sealer based on tricalcium silicate, which is a hydrophilic sealer that requires water presence to set and harden. The setting time is 25 min, measured according to ISO 6876:2012 (100% humidity conditions). However, in normal root canals, the setting time can be more than 2.5 h as reported by the manufacturer. To our knowledge, no information has been published in scientific literature regarding the angiogenic capacity of this root canal sealer.

Our main objective was, therefore, to compare the *in vitro* cellular angiogenic responses of human dental pulp stem cells (hDPSCs), human periodontal ligament stem cells (hPLSCs), and human tooth germ stem cells (hTGSCs) when exposed to ProRoot MTA, Biodentine, and Well-Root ST. A second objective was to show the vascular effects of these materials on human umbilical vein endothelial cells (HUVECs) using the Matrigel-based tube formation assay. Three null hypotheses were tested: (1) The angiogenic response of hDPSCs, hPLSCs, and hTGSCs after their exposure to tricalcium silicate-based cements is not different; (2) Well-Root ST, Biodentine, and ProRoot MTA are equally adept at eliciting an angiogenic response in hDPSCs, hPLSCs, and hTGSCs; and (3) The vascular effects of the tested tricalcium silicate-based cements on HUVECs are not different.

## Methodology

The research protocol of this study was approved by the Istanbul Medipol University Ethical Board of Clinical Trials & Non-Interventional Research (Approval Number:10840098-604.01.01-E21424/257). Signed informed consent was obtained from all patients prior to the collection of the periodontal ligament, tooth germ, and pulp samples. The cells were obtained from healthy 15 to 25-year-old patients.

### Sample preparation

**Figure 1:**
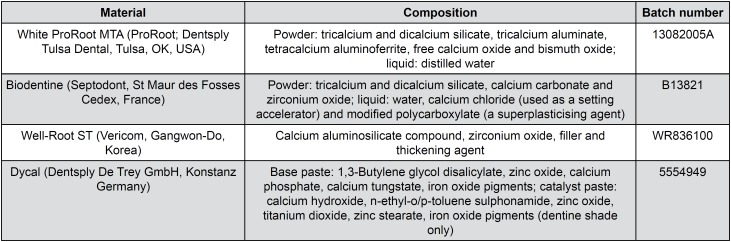
Composition description and batch numbers of the tested materials

The materials used were white ProRoot MTA (Dentsply, Tulsa Dental, Tulsa, OK), Biodentine, Well-Root ST, and Dycal (Dentsply De Trey GmbH, Konstanz, Germany). Figure 1 shows the compositions of these materials, which were prepared according to the manufacturers’ instructions: ProRoot MTA was prepared by mixing the powder and water at a ratio of 3:1, Biodentine was mixed in a high-speed amalgamator for 30 s, and Dycal was dispensed as equal volumes of base and catalyst pastes on a parchment paper pad. Well-Root ST was supplied by the manufacturer in pre-mixed syringes and required no preparation before use. The samples were prepared in a laminar flow hood under aseptic conditions and were dispensed into pre-sterilized Teflon molds (5 mm diameter and 3 mm thickness) using an MTA carrier (Dentsply Tulsa Dental, Tulsa, OK, USA). The samples could set for three days at 37°C in 80% humidity in a cell culture incubator and were then sterilized by ultraviolet light for 4 h on each surface. Discs of tricalcium silicate-based cements and Dycal were immersed in Dulbecco's modified Eagles’ medium (DMEM) for 24 h. A medium that had not been treated with the cement materials served as a negative control for ELISA, tube formation assays, and MTS assays, and a medium treated with Dycal was used as a positive control for the tube formation and MTS assays.

### Isolation of hTGSCs, hDPSCs, and hPDLSCs and cell culture conditions

The hTGSCs, hDPSCs, and hPLSCs were isolated and characterized as described previously;^16^^–^^18^ The hTGSCs were collected from the mandibular third molar tooth, and the hDPSCs and hPDLSCs were collected from maxillary second premolar teeth of 15 to 25-year-old healthy patients. The collected tissues were harvested, minced, and plated in six-well plates (BIOFIL, TCP, Switzerland). Cells were grown in DMEM supplemented with 10% (v/v) fetal bovine serum (FBS) and 1% (v/v) PSA (10,000 units/mL penicillin; 10,000 μg/mL streptomycin; and 25 μg/mL amphotericin B) (Invitrogen, Gibco, UK). Once the cells had reached confluency, they were trypsinized with 0.25% (v/v) trypsin/EDTA (Invitrogen) and seeded in a T75 flask (Zelkultur Flaschen, Switzerland). The cells were maintained at 37°C and 5% CO_2_ in a humidified incubator.

### Flow cytometry-based mesenchymal stem cell characterization

Isolated hTGSCs, hDPSCs, and hPDLSCs (passage 3) were characterized for their mesenchymal cell surface profiles, as described previously.^16^^–^^18^ The hTGSCs and hDPSCs were trypsinized and incubated with the following conjugated antibodies: CD29, CD34, CD45, CD73, CD90, CD105, CD133, and CD166 (Santa Cruz Biotechnology Inc., Santa Cruz, CA, USA). The hPDLSCs were then incubated with primary antibodies raised against STRO-1, CD146, CD90, CD44, CD19, or CD14. The cells were washed with phosphate-buffered saline (PBS) to remove the excess primary antibodies. The cells were analyzed by flow cytometry using a FACSCalibur flow cytometry system (Becton Dickinson, San Jose, CA, USA).

### MTS cell viability assays

Cell viability was measured on the 1^st^, 3^rd^, 7^th^, 10^th^, and 14^th^ days, using the 3-(4,5-dimethyl-thiazol-2-yl)-5-(3-carboxy-methoxy-phenyl)-2-(4-sulfo-phenyl)-2H tetrazolium (MTS) assay (CellTiter96 Aqueous One Solution, Promega, UK). For each material group and for each time point, 100 µL of sample-treated medium was added to transwell inserts (Corning, NY, USA) and co-cultured with hTGSCs, hPDLSCs, and hDPSCs (20,000 cells *per* well) in 24-well plates. The cells in media were incubated for 14 days, and the media were changed every other day. On the evaluation days, MTS solution was prepared according to the manufacturer's instructions, and the cells were treated with this solution. The plates were incubated for 2 h in the dark, and then 100 μL of sample from each well was transferred to 96-well plates. The tricalcium silicate-based cements toxicity effects on cell viability were determined by measuring absorbance at 490 nm with an ELISA plate reader (Biotek, USA).

### Tube formation assay

A Matrigel-based tube formation assay was performed as described previously to show the vascular effects of tricalcium silicate-based cements.^19^ Frozen growth factor-reduced Matrigel (BD Bioscience) was warmed up to room temperature, and 150 μL was plated onto 48-well plates on ice so that Matrigel covered the plate surface, and the plates were incubated for 30 min at 37°C. Human umbilical vein endothelial cells (HUVECs) were used to determine the angiogenic potential of the cement discs. HUVECs were purchased from ATCC (ATCC^®^ CRL-1730™) and cultured in DMEM, supplemented with 10% fetal bovine serum (FBS; Hyclone, UT, USA) in a humidified incubator at 37°C with 5% CO_2_. The HUVECs were seeded on Matrigel-coated plates (100,000 cells/50 μL) with serum-free high-glucose DMEM as control. For each material group, the medium was added onto cement discs containing transwell inserts (Corning, NY, USA) and co-cultured with HUVECs. Following incubation at 37°C for 6–8 h, each well was analyzed directly under an inverted light microscope. Tube formation in each field was imaged, and an average of tubules from five random fields in each well was counted.

### Enzyme-linked immunosorbent assays

FGF-2, VEGF, and PDGF protein levels were measured in the culture medium from tricalcium silicate-based cement-treated hTGSCs, hDPSCs, and hPDLSCs by sandwich ELISA performed with kits (PeproTech, USA). Protein standards and samples were added to the antibody-coated plates and incubated overnight at 4°C. Biotinylated detection antibodies were added to the plates and incubated at room temperature for 2 h, followed by incubation with peroxidase-labelled streptavidin and tetramethylbenzidine (TMB) substrate.

### Statistical analysis

The findings were registered in a Microsoft Excel (2010) spreadsheet and the statistical evaluation was performed using IBM SPSS Statistics 22 (IBM Corporation, NY, USA). The Shapiro-Wilk's test was used to confirm the assumption of data normality. The variables with normal distributions were expressed as the mean and standard deviation (mean ± SD), and those without normal distributions were expressed as the median. The Kruskal-Wallis test was used in the comparison between the groups, and the Mann-Whitney U test was used to determine the group causing the difference, because the data did not show a normal distribution. The variances homogeneity was determined using Levene's test. The Friedman test was used for intra-group comparison of the parameters, and the Wilcoxon signed rank test was used as the *post-hoc* test. The signiﬁcance was set at the 95% conﬁdence level.

## Results

### Cell attachment and viability

**Figure 2:**
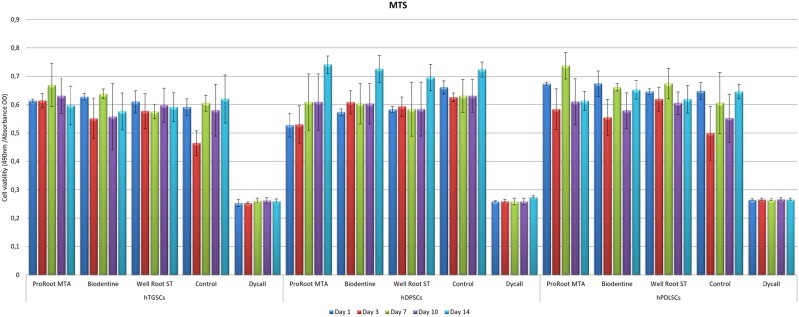
Tested materials effects on MTS results. Cell viability was evaluated by MTS assay at the 1^st^, 3^rd^, 7^th^, 10^th^ and 14^th^ days. hDPSCs: human dental pulp stem cells; hPLSCs: human periodontal ligament stem cells; hTGSCs: human tooth germ stem cells (Significant: *p<0.05)

Figure 2 shows the MTS values within and between the groups of individual materials in the different cell groups.

In the hTGSCs group, cell viability was significantly enhanced by Well-Root ST, Biodentine, and ProRoot MTA when compared with Dycal and the control groups at day three (p<0.05). However, on the first, seventh, tenth and fourteenth day, the MTS levels in all groups showed no statistically significant differences except for the Dycal group, which showed lower cell viability (p>0.05).

The hDPSCs group showed the following statistically significant differences in cell viability between the materials based on the first-day MTS values: Control>Well-Root ST=Biodentine>ProRoot MTA>Dycal (p<0.05). On the third day, the MTS level was significantly lower for the Dycal group than for the ProRoot MTA, Biodentine, Well-Root ST, and control groups (p<0.05). The third-day MTS level of the controls was found to be significantly higher than in the ProRoot MTA group (p<0.05).

The hPLSCs group showed significantly higher first-day MTS values for the ProRoot MTA group than for the Well-Root ST group (p<0.05). No statistically significant differences were noted for the other materials (p>0.05). The third-day MTS values were significantly higher for the Well-Root ST group than for the control group (p<0.05). No statistically significant difference was noted for the other materials (p>0.05). On the seventh day, the MTS values were significantly higher for the ProRoot MTA group than for the Biodentine and control groups (p<0.05).

### ELISA

**Figure 3:**
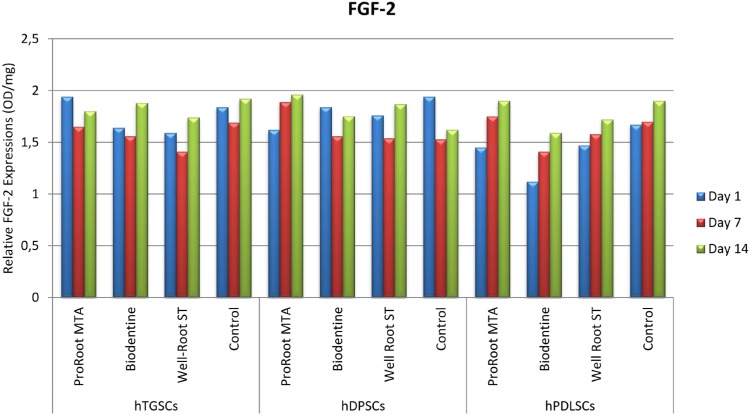
The visualized results of tested materials with ELISA test on different stem cell types for FGF-2. hDPSCs: human dental pulp stem cells; hPLSCs: human periodontal ligament stem cells; hTGSCs: human tooth germ stem cells; FGF-2: basic fibroblast growth factor 2

**Figure 4:**
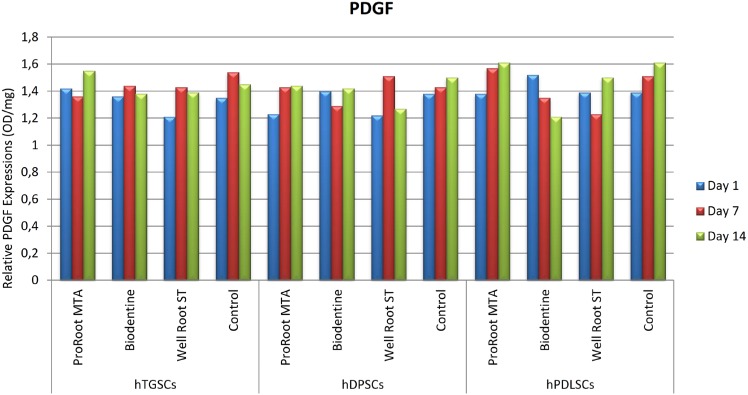
The visualized results of tested materials with ELISA test on different stem cell types for PDGF. hDPSCs: human dental pulp stem cells; hPLSCs: human periodontal ligament stem cells; hTGSCs: human tooth germ stem cells; PDGF: platelet-derived growth factor

**Figure 5:**
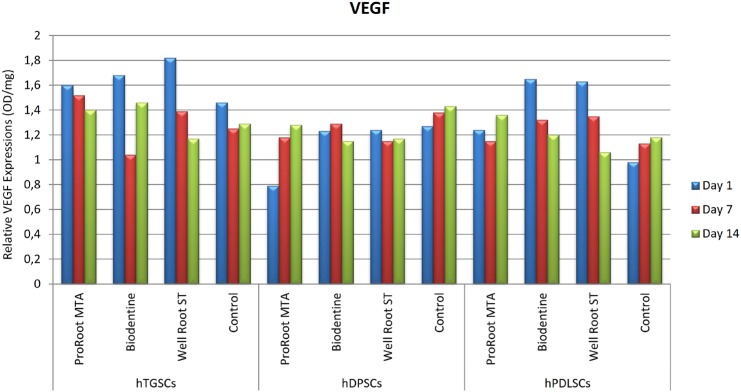
The visualized results of tested materials with ELISA test on different stem cell types for VEGF. hDPSCs: human dental pulp stem cells; hPLSCs: human periodontal ligament stem cells; hTGSCs: human tooth germ stem cells; VEGF: vascular endothelial growth factor

The hTGSC, hDPSC, and hPLSC groups showed no statistically significant differences in FGF-2, PDGF, and VEGF levels between the first, seventh, and fourteenth days when exposed to the test materials (p>0.05). The ELISA results for FGF-2, PDGF, and VEGF are shown in Figure 3, 4, and 5, respectively, for the various cell types. The results did not show statistical significance, but the hTGSCs showed higher VEGF levels on the first and seventh days, and especially on the first day, in response to Well-Root ST when compared to the other test materials (p>0.05). At the fourteenth day, the VEGF levels were higher in the ProRoot MTA and Biodentine groups than in the Well-Root ST and control groups (p>0.05). In the hPLSCs, the VEGF levels were higher in the Biodentine and Well-Root ST groups at the first and seventh days, and especially at first day, but at the fourteenth day, the VEGF levels were higher for ProRoot MTA than for the other tested materials, although the difference did not reach statistical significance (p>0.05). The highest FGF-2 levels were obtained with Biodentine in hTGSCs and with Well-Root ST and ProRoot MTA in hDPSCs, although the differences did not reach statistical significance (p>0.05).

### Tube formation assay

**Figure 6:**
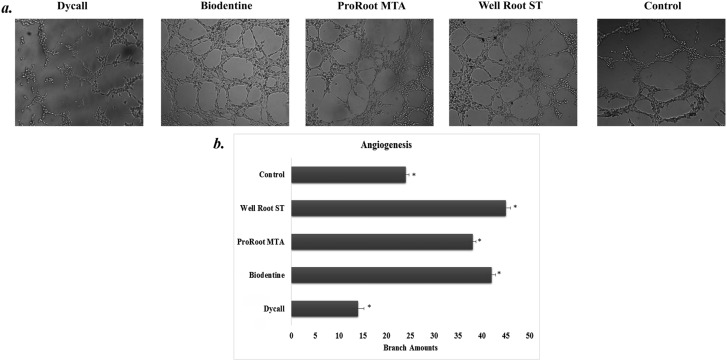
(a) Tubular network formation of HUVEC cells on Matrigel. Representative images showing tubular network formation at 6-8 hours after cell seeding (100,000 cell/50 μL with serum-free DMEM). The groups showed tubular network formation as follows, respectively: Well-Root ST>Biodentine>ProRoot MTA>Control>Dycal (p<0.05). HUVECs: human umbilical vein endothelial cells; DMEM: Dulbecco's modified Eagle's medium. (b) Quantitative analysis of angiogenic tubule formation. Comparison of parameters of tube formation assay among tested materials (Significant: *p<0.05)

Statistically significant differences were noted in terms of tubular network formation by HUVECs, as follows: Well-Root ST>Biodentine>ProRoot MTA>Control>Dycal (p<0.05) (Figure 6A, 6B).

## Discussion

In this study, due to the known contributions of PDGF, FGF-2, and VEGF to angiogenesis, their release was evaluated from odontogenic stem cells exposed to the tricalcium silicate-based materials Well-Root ST, Biodentine, and ProRoot MTA.^6^ Untreated cells were used as a negative control group, and Dycal served as a positive control group because Dycal was previously shown to be cytotoxic to hTGSCs.^20^

The MTS results showed no statistically significant difference between the hTGSC groups on the first day, but the cell viability was higher on the third day in the Well-Root ST, Biodentine, and ProRoot MTA groups than in the positive control group. In the hDPSC groups, a statistically higher cell viability level was observed on the first day for the Well-Root ST group than for the ProRoot MTA and Dycal groups. In the hPLSC groups, a statistically higher viability was observed in the Well-Root ST group than in the positive control group on the third day. The Well-Root ST group also showed stable results at all time points in hPLSCs, although the cell viability varied in the ProRoot MTA and Biodentine groups during the experiment.

Our findings indicate that Well-Root ST, which is a newly developed tricalcium silicate-based material, is a suitable alternative to ProRoot MTA and Biodentine in terms of its potential for enhancing angiogenesis. Its effectiveness is likely due to its similar chemical components, as stated by the Well-Root ST manufacturer. The use of ProRoot MTA material in hDPSCs resulted in no significant change between the third day and the first day, but the increases in cell viability on the seventh, tenth, and fourteenth days were statistically significant. Conversely, the Well-Root ST treated hDPSCs showed no significant differences at any day, while the Biodentine group showed statistically significant increases on the fourteenth day. After the 14-day period, all the tested materials were deemed biocompatible and all had good bioactivity, as indicated by cell growth induction. The high cell viability of the Well-Root ST treated group on the first day indicates a high probability of successful guided endodontic repair with this material. These findings are consistent with previous findings by Costa, et al.^12^ (2016) and Peters, et al.^21^ (2016), who demonstrated that ProRoot MTA and Biodentine had similar good cell viability. Another study also indicated that ProRoot MTA elicited great cell viability, determined by the MTS assay.^22^ These results were also confirmed by Chung, et al.^23^ (2016), who demonstrated that cells exposed to ProRoot MTA were well attached, with no inhibition zone observed around the cement at either day three or day seven.

The ELISA results also indicated no differences between the cell groups and the tested materials in terms of angiogenic-enhancing potential. A possible explanation for this effect could be differences in the test materials; that is, preparation of the cements in static conditions versus using extracts from set materials. Set materials in static conditions were used to recapitulate the long-term clinical conditions according to the manufacturers’ instructions.^16^^,^^24^^,^^25^ Consequently, the effect of the tested materials on cell behavior was similar and minor. Chung, et al.^23^ (2016) found that VEGF levels were significantly higher in a ProRoot MTA group than in a control group, but no difference was found between the groups for FGF-2 levels. Contrary to this findings, Paranjpe, et al.^26^ (2010) reported a significant increase in the VEGF secretion from human dental pulp stem cells in response to ProRoot MTA, whereas no difference was found in this study among the ProRoot MTA, Biodentine, and Well-Root ST groups.

Although the extracts from the tested cements did not significantly affect cell responses (viability and growth factor release), some tendencies are worth mentioning. The Well-Root ST and Biodentine groups of hPDLSCs and hTGSCs released higher VEGF levels at the first and third days, and especially at the first day. The difference in the results of this study and the previous one by Paranjpe, et al.^26^ (2010) could be related to the use of cells treated with 20 mmol/L N-acetyl cysteine as the control group in the previous study,^26^ whereas the our control group was untreated cells. A possible explanation for this effect could be that Well-Root ST and Biodentine at early stages after setting have a higher ion-releasing potential, which would directly affect the cell interaction. Our findings are consistent with those by Peters, et al.^21^ (2016), who showed that ProRoot MTA and Biodentine stimulated the expression of angiogenic genes and the release of VEGF and induced similar expression patterns.

Herein, HUVECs were used to show angiogenic potential by the tube formation assay, which is recognized as a basic vascularization model for *in vitro* studies.^12^^,^^22^^,^^27^^,^^28^ The tube formation assay results revealed that Well-Root ST, Biodentine, and ProRoot MTA all enhanced the HUVECs angiogenic potential. Well-Root ST showed the best angiogenic response, which might be related to the higher tendency of cells treated with Well-Root ST to release VEGF, an essential factor for the vascular system differentiation at the first day in all cell types. In contrast to these results, Chang, et al.^27^ (2015) reported that ProRoot MTA induced a significant increase in the expression of angiogenic genes and in capillary tube formation. Costa, et al.^12^ (2016) also showed that ProRoot MTA and Biodentine were not different in their abilities to increase HUVEC growth.

Our search in literature revealed only a limited number of studies using Well-Root ST, and no study has yet examined the angiogenic-enhancing potential of this bioceramic endodontic sealer. These results provide significant information that could guide clinicians in selecting alternative materials for interaction with various cell types to increase the success of guided endodontic repair therapies. A better understanding on the role of endodontic bioceramic cements on different cell types regarding the angiogenic growth factors released by the pulp to support periapical tissue regeneration and the identification of possible mechanisms for enhancing angiogenesis with promising materials, such as Well-Root ST, Biodentine, or ProRoot MTA, should be among the goals of future research.

## Conclusions

Altogether, and within the limitations of this *in vitro* study, the results from the tube formation assays indicate that Well-Root ST can stimulate better angiogenesis and new vessel formation during endodontic regeneration procedures than is achieved with either Biodentine or ProRoot MTA. The results also indicated that ProRoot MTA, Biodentine, and Well-Root ST had similar biological effects on hDPSCs, hPLSCs, and hTGSCs, whereas Dycal demonstrated specific cytotoxicity, according to ELISA results. This study highlights the significance of using ProRoot MTA, Biodentine, and especially Well-Root ST as effective and appropriate agents in regenerative endodontic therapies. In addition, due to the characteristic properties of the materials, an important point to remember is that they elicit different responses under varying conditions. Therefore, future studies should focus on the variety of material forms and/or environments effect on stem cell behavior with the objective of contributing to biological aspects in the regenerative literature.
